# Are Ionic Liquids Better Extracting Agents Than Toxic Volatile Organic Solvents? A Combination of Ionic Liquids, Microwave and LC/MS/MS, Applied to the Lichen *Stereocaulon glareosum*

**DOI:** 10.3389/fchem.2020.00450

**Published:** 2020-05-29

**Authors:** Erika Calla-Quispe, Juana Robles, Carlos Areche, Beatriz Sepulveda

**Affiliations:** ^1^Instituto de Ciencias Ómicas y Biotecnología Aplicada, Pontificia Universidad Católica del Perú, Lima, Peru; ^2^Departamento de Química, Facultad de Ciencias, Universidad de Chile, Santiago, Chile; ^3^Departamento de Ciencias Químicas, Universidad Andrés Bello, Viña del Mar, Chile

**Keywords:** lichens, ionic liquids, LC/MS, natural products, stereocaulon

## Abstract

We report a green strategy for the extraction of lichen substances from *Stereocaulon glareosum*. This sustainable alternative does not use volatile toxic organic solvents, but it is assisted by microwave and is checked by UHPLC/ESI/MS/MS. Ionic liquids may provide a better alternative in the extraction of natural products from lichens.

**Graphical Abstract:**
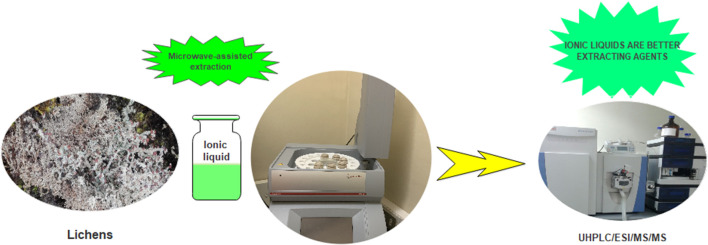


## Introduction

Traditionally, most organic chemists routinely use volatile organic compounds (VOCs) in both synthetic processes, and in extraction techniques, to obtain organic extracts, such as maceration, infusion, distillation, percolation, and Soxhlet. In this sense, many natural product chemists have long attempted to change the se traditional methods by green ones; e.g., microwave, ultrasound, pressurized solvents, pulsed electric field, and high speed homogenization; for the extraction of secondary metabolites (Chemat et al., 2012; Ibañez and Cifuentes, 2017; Soquetta et al., 2018; Chatel and Varma, 2019).

Natural product processing, in the extraction, isolation and purification of these metabolites, implies the use of organic solvents, both in academia and in chemical industries. According to the European Union, VOC is any organic compound whose boiling point ranges up to 482°F (250°C). This hazardous material causes trouble in the pharmaceutical and chemical industry, where its extraction process is considered “dirty” when compared with perfume industries. As a result, specialists believe that its environmental impact is greater than they imagined (Chemat and Vian, 2014). A clear example of this paradigm change in organic synthesis was the redesigning of sertraline green synthesis, developed by Pfizer, obtaining a Presidential Green Chemistry Challenge Award in 2002 (Sheldon, 2010).

Solvents in organic chemistry are used during the extraction process and removed in large quantities afterwards. An accurate selection of a solvent is crucial to minimize the impact cost, safety, health, and environment. Green solvents are continuously being developed to substitute hazardous solvents by others with better health, safety, and environmental properties. As an alternative to toxic volatile organic solvents, ionic liquids (ILs), supercritical fluids, subcritical fluids, deep eutectic solvents have been proposed (Chemat and Vian, 2014). ILs are ionic species (organic salts), fluids or solids at room temperature. They consist of an organic cation (e.g., ammonium, imidazolium, pyridinium, phosphonium) and an anion (e.g., bromide, chloride, tetrafluoroborate, hexafluorophosphate). Besides, these ILs are attractive as solvents for green extraction and synthesis due to their unique properties, such as water solubility, low nucleophilicity, low vapor pressure, and a high level of extraction for organic compounds. Therefore, these features are continuously being considered by natural product researchers to extract and isolate secondary metabolites from plants. Compounds, such as flavonoids, alkaloids, phenolics, terpenoids, phenylpropanoids and polysaccharides, continue to prove the effectiveness of ILs as a green method for the extraction of these metabolites (Ventura et al., 2017; Zhao et al., 2018).

Lichens are the result of the symbiotic interaction between an ectomycorrhizic fungus (known as the mycobiont) and a photosynthetic alga (the photobiont), forming ecologically and evolutionary discrete thallus (the holobiont). The photobionts are algae present in these different groups of organisms: cyanobacteria, green algae (Chlorophyta) and brown algae (Heterokontophyta). Through time, lichens have been used for various purposes, in particular as dyes, perfumes and remedies in folk medicine. Lichens occur in some of the most extreme environments on Earth, hot deserts, rocky coasts, and toxic slag heaps. Besides, they are abundant as epiphytes in the environment; on leaves and branches in rain forests and temperate woodlands; on bare rock, including walls and gravestones; on exposed soil surfaces; and also on tiles and rooftops. Lichens are widespread, due to their poikhilohydric strategies. They are vulnerable to environmental disturbances, such as sulfur dioxide, hydrogen fluoride and other organopollutants. Species of *Evernia, Peltigera, Parmelia, Cladonia, Rocella*, and *Pertusaria*, have been used to control fever, diarrhea, infections, skin diseases, epilepsy, convulsions, and as purgative. *Peltigera canina* is a tonic used to treat liver ailments because of its high methionine content. Nowadays, the antibiotic aspects of lichens have greatly enhanced its medicinal importance (Lakatos, 2011).

Lichens have a varied chemistry and produce many compounds, including phenolic com- pounds, dibenzofurans, depsides, depsidones, depsones, lactones, anthraquinones, and pulvinic acid derivatives. Lichens present a multiple biological activity e.g., antibiotic, antitumor, antiviral, allergenic, gastroprotective, antiulcer, antiherbivore, antileishmanial, anti-inflammatory, antioxidants, antitrypanosoma, enzyme inhibitory, and plant growth inhibitory (Shukla et al., 2010; Muller, 2011; Nguyen et al., 2013; Calcott et al., 2018). Studies of the chemistry of lichens have identified more than 1,000 secondary metabolites, known as lichen substances. These chemicals are usually insoluble in water, but they can be extracted by means of organic solvents. This extracted amount ranges from 0.1 to 10% of the dry weight of the thallus, and sometimes up to 30% (Shukla et al., 2010; Muller, 2011).

The aim of this work is to provide alternative solvents for extraction of natural products from lichens. In this context, we studied extraction methods from *Stereocaulon glareosum* using ionic liquids instead of classical organic solvents, such as methanol. Finally, each extract was checked by using UHPLC/ESI/ MS/MS.

## Materials and Methods

### Chemicals

UHPLC-MS solvents were purchased from Merck (Santiago, Chile). Ultrapure water was obtained from a Millipore water purification system (Milli-Q Merck Millipore, Chile). Some standards were purchased either from Sigma Aldrich (Saint Louis, Mo, USA), or Extrasynthèse (Genay, France).

### Lichen Material

*Stereocaulon glareosum* were collected in the snowy Huaytapallana, Junin, Perú in January 2017 at 5000 m.a.s.l. (11° 57′ 15,50″S; 75° 2′ 33,77″W). The samples were identified by the lichenologist Angel Ramirez. The voucher herbarium specimens were kept at the Natural History Museum of the Universidad Nacional Mayor de San Marcos under reference number USM 278427.

### Maceration Extraction

The powdered sample of *Stereocaulon glareosum* (0.200 g) was placed in 10 mL of methanol at room temperature, and extracted for 24 h. After centrifugation (9,000 g, 30 min), the supernatant was concentrated in vacuum to yield 18 mg of a dark extract (9%).

### Ultrasound-Assisted Extraction

The powdered sample of *S. glareosum* (0.200 g) was placed in 10 mL of the following ionic liquids: 1-butyl-3-methylimidazolium methylsulfate ([Bmim]MeSO_4_); 1-butyl-3-methylimidazolium tetrafluoroborate ([Bmim]BF_4_); 1-butyl-3-methylimidazolium bromide ([Bmim]Br), and 1-butyl-3-methylimidazolium chloride ([Bmim]Cl). The ultrasound-assisted extraction of the samples was performed in an ELMA ultrasonic bath (ELMA, GmbH, Germany) at 40 kHz frequency for 30 min. After extraction, the supernatant was centrifuged for 30 min at 9,000 g, and then, filtered to induce a precipitate by using water. Unfortunately, no precipitate was obtained.

### Microwave-Assisted Extraction

The powdered samples of *S. glareosum* (0.200 g) were placed in 10 mL of the following ionic liquids: 1-butyl-3-methylimidazolium methylsulfate ([Bmim]MeSO_4_); 1-butyl-3-methylimidazolium tetrafluoroborate ([Bmim]BF_4_); 1-butyl-3-methylimidazolium bromide ([Bmim]Br), and 1-butyl-3-methylimidazolium chloride ([Bmim]Cl). Then, they were placed in a microwave device (Anton Parr, Switzerland) with adjustable operating parameters (100°C, 30 min and 10 W). After centrifugation for 30 min at 9,000 g, the supernatant was precipitated, using water (5 mL), to yield a dark gummy extract.

### UHPLC-PDA-MS Instrument

The Thermo Scientific Dionex Ultimate 3000 UHPLC system hyphenated with a Thermo Q exactive focus machine used was already reported. For the analysis, 2 mg of each extract were first dissolved in 2 mL of ethanol, then filtered (PTFE filter) and finally 10 μL were injected in the instrument, with all specifications set as previously reported (Salgado et al., 2017; Torres-Benitez et al., 2017).

#### LC Parameters and MS Parameters

Liquid chromatography was performed using an UHPLC C18 column (Accucore, 150 mm × 4.6 mm ID, 2.5 μm, Thermo Fisher Scientific, Bremen, Germany) operated at 25°C. The detection wavelengths were 254, 280, 330 and 354 nm, and PDA was recorded from 200 to 800 nm for peak characterization. Mobile phases were 1% formic aqueous solution (A) and 1% formic acid in acetonitrile (B). The gradient program (time (min), % B) was: (0.00, 12); (5.00, 12); (10.00, 20); (15.00, 40); (20.00, 40); (25.00, 70); (35.00, 12); and 15 min for column equilibration before each injection. The flow rate was 1.00 mL min^−1^, and the injection volume was 10 μL. The standards, and the extracts dissolved in ethanol, were kept at 10°C during storage in the autosampler. The HESI II and Orbitrap spectrometer parameters were optimized as previously reported (Salgado et al., 2017; Torres-Benitez et al., 2017).

## Results and Discussion

**Figure 1:**
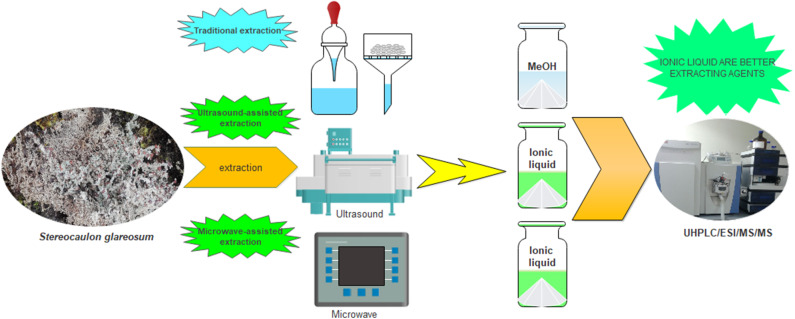
Scheme used in the extraction process.

We started with the extraction by maceration and ultrasound, two traditional common procedures in Natural Product Chemistry in order to obtain the best lichen extract yields after precipitation with water (Figure 1). According to our results, the use of maceration and ultrasound in ILs was not suitable since it did not render extracts.

This failure could be explained by the high viscosity of ILs I-IV, which avoids transfer of the compounds toward the solvent in the case of maceration. While in the case of ultrasound assisted extraction, the power could have been low (50 W at a frequency of 40 KHz). Therefore, microwave-assisted extraction was performed in duplicate, thus obtaining a more effective amount than MeOH traditional extraction. In order to check that the ILs (I-IV) are preferable extraction systems for the secondary metabolites, an untargeted metabolomics study based on UHPLC/ESI/MS/MS was performed to identify all compounds (Cornejo et al., 2016; Salgado et al., 2017; Torres-Benitez et al., 2017).

The extraction results by using the [Bmim]MeSO_4_, [Bmim]BF_4_, [Bmim]Br, [Bmim]Cl, and MeOH were by 17, 15, 14, 11, and 9% respectively. As shown, the extraction yields for ILs (I-IV) were higher than the MeOH extract. Among them, [Bmim]MeSO_4_ was the highest one, indicating that [Bmim]MeSO_4_ is more efficient for the extraction of metabolites than traditional methanol. These results could be explained by the temperature being 100°C, which reduces the viscosity of ILs, making the system thermodynamically stable, and facilitating the mass transfer process (Ventura et al., 2017). In relation to liquid/solid ratio (L/S) on the extraction efficiency, we selected an L/S ratio by 10/0.2 (mL/g) based on our experience, since L/S above of 50 mL/g do not increase the extraction yields.

**Figure 2:**
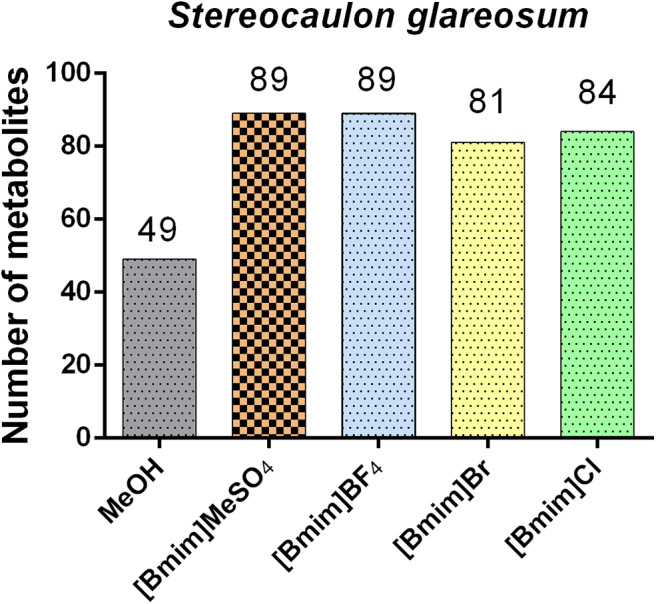
Distribution of the number of lichen substances extracted by methanol and ionic liquids.

To further evaluate the effects of ILs on the extraction efficiency, we compared all chromatograms and total ion currents for the five extracts obtained. Qualitative analysis by UHPLC/ESI/MS/MS helped to identify the chemical cluster s and the potential of each extract of *Stereocaulon glareosum*. Structural diversity was grouped as follows: depsides, depsidones, diphenylethers, dibenzofurans, lipids, monoaromatics, polyols and unknown compounds. As shown in Figure 2, the results showed that the [Bmim]MeSO_4_ and [Bmim]BF_4_ extracts were the solvents with the highest number of extracted compounds than with methanol. Even, the four ILs used in this study were more efficient than methanol. Untargeted metabolomics tentatively identified 49 compounds from the methanolic extract, and 89, 89, 81 and 84 metabolites from [Bmim]MeSO_4_, [Bmim]BF_4_, [Bmim]Br, and [Bmim]Cl extracts, respectively. In relation to mass transfer processes, it is well-known that temperature increase generally improves extraction efficiency. Besides, it could produce chemical artifacts.

**Figure 3:**
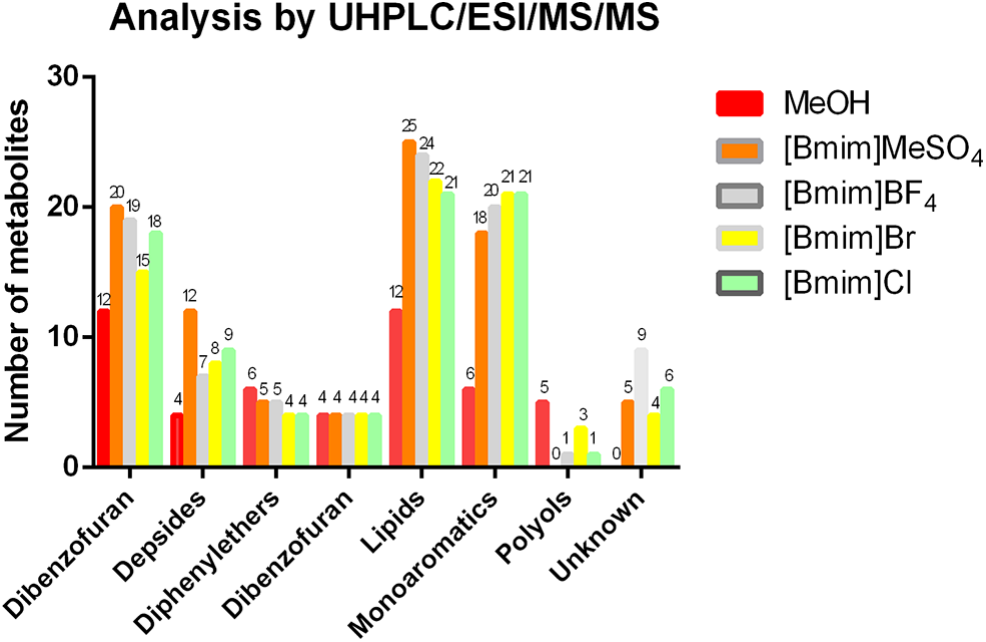
Distribution of the chemical groups extracted.

As shown in Figure 3, the qualitative analysis by the widely used UHPLC/ESI/MS/MS was performed for the characterization of secondary metabolites in complex mixtures based on their MS/MS fragmentation patters (Cornejo et al., 2016; Salgado et al., 2017; Torres-Benitez et al., 2017). On the MeOH extract, seven chemical families were detected and identified under the previously proposed LC/MS/MS analysis conditions, while on the ILs, eight families were detected, and seven identified. In the case of the family molecular of depsidones, the solvent [Bmim]MeSO_4_ extracted 20 compounds, being more efficient than methanol organic solvent (12 depsidones). In relation to the family of depsides, the solvent [Bmim]MeSO_4_ extracted 12 metabolites, being more efficient than methanol, which extracted 4 depsides. Among the solvents studied, no difference was observed in the case of the dibenzofurans extraction. In the case of the molecular family of lipids, the solvent [Bmim]MeSO_4_ extracted 25 metabolites, while methanol solvent identified 12 lipids, showing a similar tendency for depside and depsidone families. In the case of monoaromatic compounds, our results indicated that the [Bmim]Br and [Bmim]Cl solvents detected 21 compounds, being the highest ones. While the methanol extract identified six compounds, being the lowest ones. In the case of the family of unknown compounds, [Bmim]BF_4_ was more efficient for the extraction of these unidentified compounds than for the other ILs. These unknown compounds were not identified since LC/MS data did not match with theoretical information existing in the literature. For the diphenylether and polyol families, the methanol solvent showed better extraction efficiency for these type of compounds than for all ILs (for details see Table S1).

Another important case study in the discovery of natural products is related to detecting new compounds due to a high repetition rate in the extraction and purification process. In this sense, the application of ILs could be an alternative tool for the extraction and isolation of lichen substances. All new compounds reported in our study were tentatively elucidated based on the UHPLC/DAD analysis, HESI-MS/MS fragmentation and biosynthetic considerations. From the methanolic extract, five new compounds were tentatively identified for the first time (Table 1). Peak 33 showed an UV absorbance at 243, 287, 326 nm, characteristic of depsidone. The [M–H]^−^ ion at HR was detected at *m/z* 431.0983, with its fragmentation evidencing daughter ions at *m/z* 387.1074, 343.1181, and 209.0452. This implied a closed ring (2–5′); the H-5 was replaced by OH moiety (supported by 209.0452 fragment); and at 2′-OH a methyl group was added. Considering that peak 33 showed similar patters to that of elatinic acid, peak 33 was identified as 5-hydroxy-2-O-methylelatinic acid. The depsidone at peak 82 was tentatively identified as dehydroxyglomellonic acid, based on the following considerations: UV at 226, 257, 316 nm, the [M–H]^−^ ion at *m/z* 453.1554 and its MS/MS fragments at *m/z* 409.1658, and 221.0823. This last fragment [C_12_H_13_O_4_]^−^ indicated the loss of OH moiety at C-2′ from glomellonic acid. Peak 103 was identified as 2″-dehydro lobaric acid due to the presence of a double bond at C-2″ of the lobaric acid. Peak 103 showed a molecular anion at *m/z* 453.1554. The MS/MS fragmentation produced ions at 409.1659, 365.1767, and 223.0972, confirming that this depsidone is similar to lobaric acid. The fragment at *m/z* 223.0972 [C_12_H_15_O_4_]^−^ implied the absence of double bond on the pentyl group at C-6′. Therefore, the double bond should be on the other pentyl group at C-6 of the peak 103. Peak 104 was identified as 1^‴^-dehydroloxodin evidenced for its high resolution MS spectrum (453.1557), MS/MS (409.1660; 379.1561; 221.0812) and UV data (217; 278; 318 nm). The main difference with loxodin was the position of a double bond at C-1^‴^, which was assigned by the presence of the fragment at m/z 221.0812 [C_12_H_13_O_4_]^−^ and biosynthetic approaches in lichen substances. Peak 111 showed a [M–H]^−^ ion at *m/z* 177.0193. This aromatic compound is closely related to hematommic acid, based on their UV data and fragmentation patterns at *m/z* 163.0395, 133.0286, 119.0493, and 105.0337. Peak 111 was tentatively identified as hematommic acid lactone.

**Table 1:** Identification of five new compounds in methanolic extract from *S. glareosum* by UHPLC-ESI-MS/MS.

**Peak**	**Tentative identification**	**[M-H]^**−**^**	**Measured mass (*m/z*)**	**Metabolite type**	**MS/MS ions (ppm)**
33	5-hydroxy-2-O-methylElatinic acid	C_21_H_19_${\text{O}}_{10}^{-}$	431.09930	D	387.10852; 343.11884
82	Dehydroxy-glomellonic acid	C_25_H_25_${\text{O}}_{8}^{-}$	453.15359	D	409.16330; 221.08230
103	2″-dehydroLobaric acid	C_25_H_25_${\text{O}}_{8}^{-}$	453.15457	D	409.16589; 365.1759 223.09723
104	1^‴^-DehydroLoxodin	C_25_H_25_${\text{O}}_{8}^{-}$	453.15518	D	409.16605; 379.15778 221.08123
111	Haematommic acid lactone	C_9_H_5_${\text{O}}_{4}^{-}$	177.01900	A	133.02881; 119.04941; 105.03373; 163.03951

*A, aromatic; D, depsidone*.

From the [Bmim]MeSO_4_, [Bmim]BF_4_, [Bmim]Br and [Bmim]Cl extracts, 12, 8, 8, and 8 new compounds were tentatively identified, respectively. From these, five were already described in the methanol extract. Details about this tentative chemical characterization is found in the Supplementary Material.

It is well-known that the type of solvents, and its physicochemical characteristics, such as: chemical and thermal stability, non-flammability, viscosity, density, surface tension, and negligible volatility, are important factors in an extraction system when determining the chemical clusters and number of compounds extracted (Ventura et al., 2017). The choice of ILs, with the same cation and different anions, allowed us to detect that inorganic anions (Br, Cl, and BF_4_) significantly increased their extraction. This could be explained by different internal interactions, such as π-π and Vander Walls interactions. In the case of anion MeSO_4_, hydrogen bonding could also be considered since it was the best solvent acting as extracting agent.

As a result, none of the ILs showed a particular trend about the specific types of lichen substances extracted. Overall, [Bmim]MeSO_4_ followed by [Bmim]BF_4_ showed the highest incidence of detected, identified, and unidentified compounds. ILs used in our study had restricted properties, such as weak hydrogen-bonding. Therefore, it is unable of dissolving polar compounds since the anion does not contain polar residues. The same cation for all ILs, varying only anions, was used. This fact was evidenced by the presence of more polyols detected in methanol extract than in ILs extracts (Chemat and Vian, 2014; Ventura et al., 2017; Zhao et al., 2018).

Bonny et al. (2011) reported for the first time the extraction of the depsidone norstictic acid using IL-MAE as extraction method and checked by HPTLC. In that study, the ILs [C_1_mim][MeSO_4_] and [C_2_mim][EthylSO_4_] showed the best extraction of norsticitic acid compared with conventional heat-reflux extraction. Finally, the authors concluded ILs are alternative solvents for the extraction of major lichen substances. According to our results, we could extend it to the identification of minority compounds when is coupled to MS/MS studies.

In the context of green processes, supercritical fluid extraction (SFE) has been used to retain the natural characteristics of products after their processing avoiding the presence of toxic organic solvents, thus increasing the market values of the final products. SFE uses a gas (typically CO_2_) that under supercritical conditions act as the non-polar solvent for extraction of natural compounds. Sometimes, it is possible modified that polarity by co-solvents such as ethanol, methanol, isopropanol, or ethyl lactate. As a result, SFE has been used for the isolation of metabolites or valuable extracts from many species. It is the case of Grape (*Vitis vinifera* L.), tomato (*Solanum lycopersicum* L.), thyme (*Thymus vulgaris* L.), eucalypt (*Eucalyptus* spp.), coffee (*Coffea* spp.), sunflower (*Heliantus annuus* L.), flax (*Linum usitatissimum*), rosemary (*Rosmarinus officinalis* L.), red pepper (*Capsicum anuum* L.), rice (*Oryza* variety), carotenoids (carrot, tomato, apricot, peach and pumpkin) and many others (De Melo et al., 2014; Lima et al., 2019). However, SFE is not economically viable due to high operational costs despite the excellent extraction properties. As alternative solvents many authors propose the use of ionic liquids or bio-based solvents for the more polar compounds such as alkaloids, terpenoids, flavonoids, saponins, phenolic compounds (Chemat and Vian, 2014; Torres-Valenzuela et al., 2020).

Finally, these results could support the use of ILs instead of traditional organic solvents in the extraction of lichen substances based on alternative solvents in Green Chemistry. The reason for this changes are: reduction in both extraction time and solvent consumption, higher compound extraction detected by untargeted metabolomics, and MAE (Chemat and Vian, 2014).

## Conclusions

A convenient, and effective untargeted metabolomics study by using ionic liquid-based microwave-assisted extraction coupled to UHPLC/DAD/ESI/OT/MS/MS successfully detected and identified lichen substances from the lichen *Stereocaulon glareosum*. Methanol was used as control for the conventional organic extraction. Comparing the four IL extracts ([Bmim]MeSO_4_, [Bmim]Br, and [Bmim]Cl and [Bmim]BF_4_) with MeOH extract, we could conclude that ILs displayed a higher compound extraction index as extracting agents. The LC/MS plan was used for the tentative structural elucidation by using both high-resolution MS/MS studies and untargeted methodologies. These techniques provided 89 [Bmim]MeSO_4_ compounds and 89 [Bmim]BF_4_ compounds, which were better solvents than the 49 MeOH compounds for the extraction from lichens. ILs assisted by microwave-radiation, as extraction methods, were simple, rapid, effective and inexpensive. Even though ILs showed high potential as sustainable solvents for screening of lichen substances, their recovery and toxicity are still being questioned.

## Data Availability Statement

All datasets presented in this study are included in the article/Supplementary Material.

## Author Contributions

BS and CA designed this study. EC-Q and JR collected the Lichen. EC-Q and BS analyzed the LC/MS data. EC-Q, CA, and BS draft preparation. All authors read and approved the final manuscript.

## Conflict of Interest

The authors declare that the research was conducted in the absence of any commercial or financial relationships that could be construed as a potential conflict of interest.
