# Supplementary material for: Are Ionic Liquids Better Extracting Agents Than Toxic Volatile Organic Solvents? A Combination of Ionic Liquids, Microwave and LC/MS/MS, Applied to the Lichen Stereocaulon glareosum
Source: Front Chem. 2020 May 29;8:450. doi: 10.3389/fchem.2020.00450 (PMC7272493; doi:10.3389/fchem.2020.00450)
Supplement: Supplementary file 1 [file Table_1.DOCX]

**SUPPLEMENTARY MATERIAL**

**Are ionic liquids better extracting agents than toxic volatile organic solvents? A combination of ionic liquids, microwave and LC/MS/MS, applied to the lichen *Stereocaulon glareosum,* could be the first example.**

**Table of Contents:**

1. Table 1. Identiﬁcation of metabolites in Peruvian lichen *Stereocaulon glareosum* by UHPLC-ESI-Q-Orbitrap-MS/MS.

2. Figure 1. Chemical structures of the compounds identified by UHPLC-ESI-Q-Orbitrap-MS/MS.

3. Characterization data for lichen substances by using UHPLC/ESI/MS/MS

4. UHPLC-ESI-Q-Orbitrap-MS/MS Chromatogram of the extracts of S. glareosum.

| **1. Table 1. Identiﬁcation of metabolites in Peruvian lichen *Stereocaulon glareosum* by UHPLC-ESI-Q-Orbitrap-MS/MS.** | | | | | | | | | |
| --- | --- | --- | --- | --- | --- | --- | --- | --- | --- |
| **Peak** | **Tentative identification** | **[M-H]^-^** | **Retention time t_R_ (min)** | **Theoretical mass (*m/z*)** | **Measured mass (*m/z*)** | **Accuracy (ppm)** | **Metabolite type** | **MS^2^ ions (ppm)** | **Lichen *S. glareosum*** |
| 1 | Mannitol | C_6_H_13_O_6_^-^ | 1.36 | 181.07176 | 181.07138 | 2.10 | P | 151.06075 | MeOH, LI-Br, LI-Cl, LI-TFB |
| 2 | Gluconic acid^a^ | C_6_H_11_O_7_^-^ | 1.39 | 195.05103 | 195.05034 | 3.54 | P | 129.01889; 151.06076; 159.02936; 177.04016 | MeOH, LI-Br |
| 3 | Arabitol | C_5_H_11_O_5_^-^ | 1.39 | 151.06120 | 151.06076 | 2.91 | P | 133.04982 | MeOH |
| 4 | Arabic acid | C_5_H_9_O_6_^-^ | 1.39 | 165.04046 | 165.03989 | 3.45 | P | 129.01880 | MeOH, LI-Br |
| 5 | 2,5-Didehydro-D-gluconic acid | C_6_H_7_O_7_^-^ | 1.50 | 191.01973 | 191.01953 | 1.05 | P | 147.02933; 129.01862 | MeOH |
| 6 | Methyl 3-hydroxy orsellinate | C_9_H_9_O_5_^-^ | 3.74 | 197.04555 | 197.04865 | 15.73 | A | 165.01860; 149.02373; 121.02882 | LI-MS, LI-TFB |
| 7 | 2,4-Dihydroxybenzoic acid | C_7_H_5_O_4_^-^ | 5.75 | 153.01933 | 153.01891 | 2.74 | A | 109.02764 | LI-MS |
| 8 | 2,4-Di-O-methyl-5-hydroxyorsellinic acid | C_10_H_11_O_5_^-^ | 7.53 | 211.06120 | 211.06418 | 14.12 | A | 167.07127; 152.04590 | LI-Br, LI-Cl, LI-TFB |
| 9 | Pentahydroxydioxoheptadecanoic acid | C_17_H_29_O_9_^-^ | 10.30 | 377.18171 | 377.18259 | 2.33 | L | 341.16019 | LI-Br |
| 10 | Benzoic acid | C_7_H_5_O_2_^-^ | 10.34 | 121.02950 | 121.02895 | 4.54 | A | - | LI-Br, LI-Cl, LI-MS, LI-TFB |
| 11 | Isostrepsilic acid | C_15_H_11_O_6_^-^ | 12.09 | 287.05611 | 287.05652 | 1.43 | DBF | 257.04565; 243.06602; 213.05804 | MeOH |
| 12 | Methyl 7-octenoate | C_9_H_15_O_2_^-^ | 12.21 | 155.10775 | 155.10733 | 2.71 | L | 123.08076 | LI-Br, LI-Cl, LI-MS, LI-TFB |
| 13 | 4,5-Dihydroxy-2-nonenoic acid | C_9_H_15_O_4_^-^ | 12.25 | 187.09760 | 187.09721 | 2.08 | L | 145.05013; 101.02357 | MeOH, LI-Br, LI-Cl, LI-MS, LI-TFB |
| 14 | 2,4-Dicarboxy-3-hydroxy-5-methoxytoluene | C_10_H_9_O_6_^-^ | 12.50 | 225.04046 | 225.04051 | 0.22 | A | 181.05017; 137.06021 | LI-MS, LI-TFB |
| 15 | Methyl orsellinate^a^ | C_9_H_9_O_4_^-^ | 13.06 | 181.05008 | 181.05003 | 0.28 | A | 149.02361; 105.03378 | LI-Br, LI-Cl |
| 16 | Demethyllobaritonic acid | C_12_H_13_O_5_^-^ | 13.06 | 237.07685 | 237.07648 | 1.56 | A | 193.08615; 175.07584 | LI-Br, LI-Cl |
| 17 | 8-acetoxyoctanoic acid | C_10_H_17_O_4_^-^ | 13.74 | 201.11323 | 201.11317 | 0.30 | L | 186.08417; 157.12309 | LI-MS, LI-TFB |
| 18 | Atranol | C_8_H_7_O_3_^-^ | 14.89 | 151.04007 | 151.03947 | 3.97 | A | 123.04450 | MeOH, LI-Br, LI-Cl, LI-MS, LI-TFB |
| 19 | Strepsilin | C_15_H_9_O_5_^-^ | 15.84 | 269.04555 | 269.04575 | 0.74 | DBF | 149.02374; 123.04458 | MeOH, LI-Br, LI-Cl, LI-MS, LI-TFB |
| 20 | Haematommic acid | C_9_H_7_O_5_^-^ | 16.38 | 195.02990 | 195.02969 | 1.08 | A | 167.03467; 151.03951; 123.04433 | LI-Br, LI-Cl, LI-MS, LI-TFB |
| 21 | 6-Methoxystrepsilin | C_16_H_11_O_5_^-^ | 16.74 | 283.06120 | 283.06152 | 1.13 | DBF | 268.03760; 225.0560 | LI-Br, LI-Cl, LI-MS, LI-TFB |
| 22 | 5'-Methoxy-2-O-methylsquamatic acid | C_21_H_21_O_10_^-^ | 16.80 | 433.11402 | 433.11343 | 1.36 | d | 389.12430; 211.06024; 167.07083; 152.04257 | LI-MS |
| 23 | Tetrahydroxynonadecanoic acid | C_19_H_37_O_6_^-^ | 17.44 | 361.25956 | 361.25845 | 3.07 | L | - | LI-Br, LI-Cl, LI-MS, LI-TFB |
| 24 | Hypoconstictic acid derivative | C_19_H_15_O_9_^-^ | 17.57 | 387.07216 | 387.07263 | 1.21 | D | 343.08234; 299.09341 | MeOH |
| 25 | Pentahydroxytricosanoic acid | C_23_H_45_O_7_^-^ | 17.67 | 433.31708 | 433.31442 | 6.14 | L | 415.30646 | LI-Br |
| 26 | Lobaritonic acid | C_13_H_15_O_5_^-^ | 17.87 | 251.09250 | 251.09230 | 0.80 | A | 207.10303 | LI-Br, LI-Cl, LI-MS, LI-TFB |
| 27 | Trihydroxyoctadecenoic acid | C_18_H_33_O_5_^-^ | 18.27 | 329.23335 | 329.23242 | 2.82 | L | 311.22229; 183.13867 | LI-MS |

| **Tabla 1. *Cont.*** | | | | | | | | | | |
| --- | --- | --- | --- | --- | --- | --- | --- | --- | --- | --- |
| **Peak** | **Tentative identification** | **[M-H]^-^** | **Retention time t_R_ (min)** | **Theoretical mass (*m/z*)** | **Measured mass (*m/z*)** | **Accuracy (ppm)** | **Metabolite type** | **MS^2^ ions (ppm)** | **Lichen *S. glareosum*** |  |
| 28 | Stictic acid | C_19_H_13_O_9_^-^ | 18.43 | 385.05651 | 385.05658 | 0.18 | D | 357.06128; 341.06677 | MeOH, LI-Br, LI-Cl, LI-MS, LI-TFB |  |
| 29 | Pentahydroxydocosanoic acid | C_22_H_43_O_7_^-^ | 18.54 | 419.30143 | 419.30042 | 2.41 | L | 401.29132 | MeOH, LI-Br, LI-Cl, LI-MS, LI-TFB |  |
| 30 | Hexahydroxyoxohexacosanoic acid | C_26_H_49_O_9_^-^ | 18.61 | 505.33821 | 505.33878 | 1.13 | L | 487.32770; 461.34842 | MeOH, LI-Br, LI-Cl, LI-MS, LI-TFB |  |
| 31 | Tetrahydroxyeicosanoic acid | C_20_H_39_O_6_^-^ | 18.62 | 375.27521 | 375.27420 | 2.69 | L | 313.20316 | MeOH, LI-Br, LI-Cl, LI-MS, LI-TFB |  |
| 32 | Pentahydroxytricosanoic acid | C_23_H_45_O_7_^-^ | 18.77 | 433.31708 | 433.31705 | 0.07 | L | 415.30753 | LI-Br, LI-Cl, LI-MS, LI-TFB |  |
| 33 | 5-hydroxy-2-O-methylElatinic acid | C_21_H_19_O_10_^-^ | 18.81 | 431.09837 | 431.09930 | 2.16 | D | 387.10852; 343.11884 | MeOH, LI-Br, LI-Cl, LI-MS, LI-TFB |  |
| 34 | Tetrahydroxyoxotricosanoic acid | C_23_H_43_O_7_^-^ | 18.98 | 431.30143 | 431.30005 | 3.20 | L | 413.28976 | LI-Cl, LI-TFB |  |
| 35 | 9-oxodecenoic acid | C_10_H_15_O_3_^-^ | 18.98 | 183.10266 | 183.10202 | 3.50 | L | 139.11220 | LI-Br, LI-Cl, LI-MS, LI-TFB |  |
| 36 | 6-O-Methylnorascomatic acid | C_16_H_13_O_5_^-^ | 19.09 | 285.07685 | 285.07672 | 0.46 | DBF | 226.06354; 255.06735 | MeOH, LI-Br, LI-Cl, LI-MS, LI-TFB |  |
| 37 | Pentahydroxytricosanoic acid | C_23_H_45_O_7_^-^ | 19.19 | 433.31708 | 433.31442 | 6.14 | L | 415.30753 | LI-Cl, LI-MS, LI-TFB |  |
| 38 | Tetrahydroxyheneicosanoic acid | C_21_H_41_O_6_^-^ | 19.25 | 389.29086 | 389.29095 | 0.23 | L | 345.22903 | MeOH, LI-Br, LI-Cl, LI-MS, LI-TFB |  |
| 39 | 5,7-Dihydroxy butylphthalide | C_12_H_13_O_4_^-^ | 19.29 | 221.08193 | 221.08163 | 1.36 | A | 177.09149 | LI-Br, LI-Cl, LI-MS, LI-TFB |  |
| 40 | Unknown | C_14_H_29_O_8_^-^ | 19.33 | 325.18679 | 325.18433 | 7.56 | - | - | LI-Cl |  |
| 41 | Pentahydroxyoxotetracosanoic acid | C_24_H_45_O_8_^-^ | 19.39 | 461.31199 | 461.31274 | 1.63 | L | 417.32233 | LI-Br |  |
| 42 | Barbatol or 2,6-Dihydroxy-4-hydroxymethylbenzaldehyde | C_8_H_7_O_4_^-^ | 19.46 | 167.03498 | 167.03387 | 6.65 | A | 139.03943 | LI-Br, LI-Cl, LI-MS, LI-TFB |  |
| 43 | Lecanoric acid | C_16_H_13_O_7_^-^ | 19.46 | 317.06668 | 317.06580 | 2.78 | D | 167.03444; 149.02379; 123.04436 | LI-MS |  |
| 44 | Dihydroxytetraoxopentacosanoic acid | C_25_H_41_O_8_^-^ | 19.53 | 469.28069 | 469.28394 | 6.93 | L | 425.28790 | LI-MS |  |
| 45 | 2'-O-Methylatranorin | C_20_H_19_O_8_^-^ | 19.64 | 387.10854 | 387.10825 | 0.75 | d | 209.08157; 177.01874; 149.02373 | LI-MS |  |
| 46 | Olivetolcarboxylic acid | C_12_H_15_O_4_^-^ | 19.64 | 223.09758 | 223.09769 | 0.49 | A | 179.10712; 108.02079 | LI-Br, LI-Cl, LI-MS, LI-TFB |  |
| 47 | Pentahydroxytetracosanoic acid | C_24_H_47_O_7_^-^ | 19.68 | 447.33273 | 447.33331 | 1.30 | L | 429.32239 | LI-Br, LI-Cl, LI-MS, LI-TFB |  |
| 48 | Pentahydroxyoxopentacosanoic acid | C_25_H_47_O_8_^-^ | 19.73 | 475.32764 | 475.32721 | 0.90 | L | 431.33710 | MeOH, LI-Br, LI-Cl, LI-MS, LI-TFB |  |
| 49 | Tetrahydroxydocosanoic acid | C_22_H_43_O_6_^-^ | 19.81 | 403.30651 | 403.30533 | 2.93 | L | 385.29611 | MeOH, LI-Br, LI-MS, LI-TFB |  |
| 50 | dehydroxyhaematommic acid | C_9_H_7_O_3_^-^ | 19.83 | 163.04007 | 163.03978 | 1.78 | A | 135.04434; 119.04924 | LI-Br, LI-Cl |  |
| 51 | Pentahydroxyoxohexacosanoic acid | C_26_H_49_O_8_^-^ | 19.88 | 489.34329 | 489.34262 | 1.37 | L | 471.33246; 445.35300 | MeOH, LI-Br, LI-Cl, LI-MS, LI-TFB |  |
| 52 | Tetrahydroxytricosanoic acid | C_23_H_45_O_6_^-^ | 20.11 | 417.32216 | 417.32025 | 4.58 | L | 399.31174 | MeOH, LI-Br, LI-Cl, LI-MS, LI-TFB |  |
| 53 | 2-pentylbenzoic acid | C_12_H_15_O_2_^-^ | 20.18 | 191.10775 | 191.10750 | 1.31 | A | 121.02874 | MeOH, LI-Br, LI-Cl, LI-TFB |  |
| 54 | Pentahydroxytetracosanoic acid | C_24_H_47_O_7_^-^ | 20.20 | 447.33273 | 447.33078 | 4.36 | L | 429.32239; 403.33493 | LI-Br, LI-Cl, LI-TFB |  |
| 55 | 6-Ethyl-6-n-pentyl-pentadecan-4,5,7,8,15-pentol-15-acetate | C_24_H_47_O_6_^-^ | 20.79 | 431.33781 | 431.33740 | 0.95 | L | 416.32086; 413.32837 | LI-Br, LI-Cl, LI-MS, LI-TFB |  |

| **Tabla 1. *Cont.*** | | | | | | | | | |
| --- | --- | --- | --- | --- | --- | --- | --- | --- | --- |
| **Peak** | **Tentative identification** | **[M-H]^-^** | **Retention time t_R_ (min)** | **Theoretical mass (*m/z*)** | **Measured mass (*m/z*)** | **Accuracy (ppm)** | **Metabolite type** | **MS^2^ ions (ppm)** | **Lichen *S. glareosum*** |
| 56 | Lobarin | C_25_H_29_O_9_^-^ | 20.93 | 473.18171 | 473.18051 | 2.54 | DE | 411.18024; 251.09181; 239.09242; 223.09705 | MeOH, LI-Br, LI-Cl, LI-MS, LI-TFB |
| 57 | Pentahydroxyhexacosanoic acid | C_26_H_51_O_7_^-^ | 20.94 | 475.36403 | 475.36404 | 0.02 | L | 457.35327 | LI-MS, LI-TFB |
| 58 | Pentahydroxyoxoheptacosanoic acid | C_27_H_51_O_8_^-^ | 21.04 | 503.35894 | 503.35580 | 6.24 | L | 485.34811; 459.37006 | MeOH, LI-Br, LI-Cl, LI-MS, LI-TFB |
| 59 | 2',5-Dimethoxyelatinic acid | C_22_H_21_O_10_^-^ | 21.14 | 445.11402 | 445.11386 | 0.36 | D | 401.12424 | LI-MS |
| 60 | methyl (E)-dodeca-9,11-dienoate | C_13_H_21_O_2_^-^ | 21.15 | 209.15470 | 209.15475 | 0.24 | L | 177.12672 | LI-Br, LI-Cl, LI-MS, LI-TFB |
| 61 | Portentol | C_17_H_25_O_5_^-^ | 21.15 | 309.17087 | 309.17056 | 1.00 | L | 291.16013; 265.18076 | LI-Br, LI-Cl, LI-MS, LI-TFB |
| 62 | Dehydroxyxy-2'-O-methylnorbarbatic acid | C_19_H_19_O_6_^-^ | 21.21 | 343.11871 | 343.11853 | 0.52 | d | 179.07085; 163.03966 | LI-Br, LI-Cl, LI-MS, LI-TFB |
| 63 | Methyl pentahydroxyoxoheptacosanoate | C_28_H_53_O_8_^-^ | 21.27 | 517.37459 | 517.37354 | 2.03 | L | 499.36295; 459.36850; 487.36545 | MeOH, LI-Br, LI-Cl, LI-MS, LI-TFB |
| 64 | Gyrophoric acid | C_24_H_19_O_10_^-^ | 21.29 | 467.09837 | 467.09604 | 4.99 | d | 317.06488; 167.03351; 149.02295; 123.04385 | LI-Br, LI-Cl, LI-TFB |
| 65 | Isolecanoric acid | C_16_H_13_O_7_^-^ | 21.37 | 317.06668 | 317.06650 | 0.57 | d | 167.03433; 149.02371; 123.04435 | LI-Br, LI-Cl, LI-TFB |
| 66 | Methyl haematommate^a^ | C_10_H_9_O_5_^-^ | 21.37 | 209.04555 | 209.04526 | 1.39 | A | 177.01906; 149.02371; 133.02867 | MeOH, LI-Br, LI-Cl, LI-TFB |
| 67 | Unknown | C_23_H_21_O_9_^-^ | 21.42 | 441.11911 | 441.11655 | 5.80 | - | 183.01236 | LI-Br, LI-Cl |
| 68 | Glomellic acid | C_25_H_27_O_9_^-^ | 21.47 | 471.16606 | 471.16357 | 5.28 | d | 237.07516; 233.08041; 193.08572 | LI-Br, LI-Cl, LI-MS |
| 69 | 2-heptyl-4,6-dimethoxy-3-methylbenzoic acid | C_17_H_25_O_4_^-^ | 21.50 | 293.17583 | 293.17441 | 4.84 | A | 263.13217; 249.18596; 234.15819 | LI-Br, LI-Cl, LI-MS, LI-TFB |
| 70 | Tetrahydroxypentacosanoic acid | C_25_H_49_O_6_^-^ | 21.57 | 445.35346 | 445.35190 | 3.50 | L | 427.34067 | LI-Br, LI-Cl, LI-MS, LI-TFB |
| 71 | Sakisacaulon A | C_24_H_29_O_7_^-^ | 21.71 | 429.19188 | 429.19025 | 3.80 | DE | 385.19995; 251.09090; 179.10663 | MeOH, LI-Br, LI-Cl, LI-MS, LI-TFB |
| 72 | 2,4-Diformyl-3,5-dihydroxytoluene | C_9_H_7_O_4_^-^ | 21.76 | 179.03498 | 179.03342 | 8.71 | A | 151.03912; 123.04484 | LI-Br, LI-Cl, LI-TFB |
| 73 | 2’-methoxyglomellic acid | C_26_H_29_O_9_^-^ | 21.82 | 485.18171 | 485.18210 | 0.80 | d | 251.09230; 233.08174; 207.10226 | LI-MS |
| 74 | 4-demethyl-2’’-dehydrolobaric acid | C_24_H_23_O_8_^-^ | 21.87 | 439.13984 | 439.13715 | 6.13 | D | 395.14795; 325.14579  223.0971 | LI-Br, LI-Cl, LI-MS, LI-TFB |
| 75 | Stenosporonic acid | C_22_H_21_O_8_^-^ | 21.93 | 413.12419 | 413.12286 | 3.22 | D | 369.13463; 217.04951; 193.05162 | LI-Br, LI-Cl, LI-MS, LI-TFB |
| 76 | Unknown | C_25_H_23_O_9_^-^ | 22.03 | 467.13476 | 467.13455 | 0.45 | - | - | LI-MS, LI-TFB |
| 77 | 4-O-Methylolivetolcarboxylic acid | C_13_H_17_O_4_^-^ | 22.06 | 237.11323 | 237.11285 | 1.60 | A | 122.03656 | LI-Br, LI-Cl, LI-MS, LI-TFB |
| 78 | Glomellonic acid | C_25_H_25_O_9_^-^ | 22.12 | 469.15041 | 469.15097 | 1.20 | D | 381.17120 | LI-Br, LI-Cl, LI-MS, LI-TFB |
| 79 | Unknown | C_26_H_53_O_8_^-^ | 22.15 | 493.37459 | 493.37598 | 2.82 | - | - | LI-MS, LI-TFB |

| **Tabla 1. *Cont.*** | | | | | | | | | |
| --- | --- | --- | --- | --- | --- | --- | --- | --- | --- |
| **Peak** | **Tentative identification** | **[M-H]^-^** | **Retention time t_R_ (min)** | **Theoretical mass (*m/z*)** | **Measured mass (*m/z*)** | **Accuracy (ppm)** | **Metabolite type** | **MS^2^ ions (ppm)** | **Lichen *S. glareosum*** |
| 80 | Epiphorellic acid II | C_26_H_31_O_9_^-^ | 22.24 | 487.19736 | 487.19492 | 5.01 | DE | 251.09216; 237.11299; 234.08488; 218.05817; 167.03430 | MeOH, LI-Cl, LI-MS, LI-TFB |
| 81 | 4-demethyllobaric acid | C_24_H_25_O_8_^-^ | 22.26 | 441.15549 | 441.15561 | 0.27 | D | 397.16455; 353.17508  193.0863 | MeOH, LI-Br, LI-Cl, LI-MS, LI-TFB |
| 82 | Dehydroxy-glomellonic acid | C_25_H_25_O_8_^-^ | 22.31 | 453.15549 | 453.15359 | 4.19 | D | 409.16330; 221.08230 | MeOH, LI-Cl, LI-MS, LI-TFB |
| 83 | Pseudepsidona 1 | C_26_H_31_O_9_^-^ | 22.39 | 487.19736 | 487.19467 | 5.52 | DE | 265.10776; 249.11238; 223.09724; 179.10696 | LI-Br, LI-Cl, LI-MS, LI-TFB |
| 84 | Lobarstin | C_25_H_27_O_8_^-^ | 22.39 | 455.17114 | 455.17123 | 0.20 | DE | 223.09737 | MeOH, LI-MS, LI-TFB |
| 85 | Colensoic acid | C_25_H_29_O_7_^-^ | 22.60 | 441.19188 | 441.19107 | 1.84 | D | 397.2019; 353.21353; 338.18851 | LI-Cl, LI-MS, LI-TFB |
| 86 | Glomelliferonic acid isomer | C_25_H_27_O_8_^-^ | 22.77 | 455.17114 | 455.17242 | 2.81 | D | 411.18161; 379.15601 | MeOH, LI-Br, LI-Cl, LI-MS, LI-TFB |
| 87 | 5’-hydroxy glomellic acid | C_25_H_27_O_10_^-^ | 22.83 | 487.16097 | 487.16202 | 2.16 | d | 233.08189; 209.08147 | LI-MS |
| 88 | Hydroxy-4-O-methylolivetoric acid | C_27_H_33_O_9_^-^ | 22.87 | 501.21301 | 501.21356 | 1.10 | d | 457.22153; 442.19516; 239.09384; 195.10185 | LI-Br, LI-Cl, LI-MS |
| 89 | Conloxodin | C_23_H_23_O_8_^-^ | 22.89 | 427.13984 | 427.13766 | 5.10 | D | 383.14774; 325.13852; 281.08047 | MeOH, LI-Br, LI-Cl, LI-MS, LI-TFB |
| 90 | Unknown | C_28_H_19_O_10_^-^ | 22.99 | 515.09837 | 515.09644 | 3.75 | - | - | LI-TFB |
| 91 | 2’’’-oxoLividic acid | C_27_H_29_O_10_^-^ | 23.00 | 513.17662 | 513.17596 | 1.29 | D | 469.18634; 425.19772; 237.0781 | LI-MS |
| 92 | Divaronic acid | C_21_H_21_O_7_^-^ | 23.11 | 385.12928 | 385.12704 | 5.82 | D | 326.11432; 341.13748; 297.14816 | LI-Br, LI-Cl, LI-MS, LI-TFB |
| 93 | Methyl sakisacaulon | C_25_H_31_O_7_^-^ | 23.22 | 443.20753 | 443.20691 | 1.40 | DE | 399.21899; 265.10794; 109.02861 | MeOH |
| 94 | Angardianic acid | C_19_H_35_O_4_^-^ | 23.39 | 327.25408 | 327.25400 | 0.24 | L | 283.26404 | LI-MS |
| 95 | 2-Heptyl-4-methylbenzoic acid | C_15_H_21_O_2_^-^ | 23.45 | 233.15470 | 233.15321 | 6.39 | A | 135.04449; 121.02875 | MeOH, LI-Br, LI-Cl, LI-MS, LI-TFB |
| 96 | Cladonioidesin | C_19_H_17_O_9_^-^ | 23.58 | 389.08781 | 389.08810 | 0.75 | d | 345.09763; 195.06587; 163.03949; 149.02379 | MeOH, LI-Br, LI-Cl, LI-MS, LI-TFB |
| 97 | 3-Hydroxycolensoic acid | C_25_H_29_O_8_^-^ | 23.61 | 457.18679 | 457.18652 | 0.59 | D | 413.19879 | LI-Br, LI-Cl, LI-TFB |
| 98 | Norcolensoic acid | C_24_H_27_O_7_^-^ | 23.65 | 427.17623 | 427.17578 | 1.05 | D | 339.19769 | LI-Br, LI-Cl, LI-MS, LI-TFB |
| 99 | 4-hydroxy-6-methylisobenzofuran-1,3-dione | C_9_H_5_O_4_^-^ | 23.67 | 177.01933 | 177.01794 | 7.85 | A | 133.02870 | LI-Br, LI-Cl, LI-MS, LI-TFB |
| 100 | 2,4-Dimethoxy-6-n -propylbenzoic acid^a^ | C_12_H_15_O_4_^-^ | 23.89 | 223.09758 | 223.09637 | 5.42 | A | 179.10712 | LI-Br, LI-Cl, LI-MS, LI-TFB |
| 101 | Glomelliferonic acid | C_25_H_27_O_8_^-^ | 23.89 | 455.17114 | 455.17206 | 2.02 | D | 411.18179; 251.09166; 223.09744; 207.10243 | MeOH, LI-MS, LI-TFB |
| 102 | Anziaic acid | C_24_H_29_O_7_^-^ | 24.03 | 429.19188 | 429.19287 | 2.31 | d | 223.09727; 205.08664; 179.10724 | MeOH, LI-Br, LI-Cl, LI-MS, LI-TFB |
| 103 | 2’’-dehydroLobaric acid | C_25_H_25_O_8_^-^ | 24.04 | 453.15549 | 453.15457 | 2.03 | D | 409.16589; 365.1759  223.09723 | MeOH, LI-Br, LI-Cl, LI-MS, LI-TFB |
| 104 | 1’’’-DehydroLoxodin | C_25_H_25_O_8_^-^ | 24.10 | 453.15549 | 453.15518 | 0.68 | D | 409.16605; 379.15778  221.08123 | MeOH, LI-Br, LI-Cl, LI-MS, LI-TFB |

| **Tabla 1. *Cont.*** | | | | | | | | | |
| --- | --- | --- | --- | --- | --- | --- | --- | --- | --- |
| **Peak** | **Tentative identification** | **[M-H]^-^** | **Retention time t_R_ (min)** | **Theoretical mass (*m/z*)** | **Measured mass (*m/z*)** | **Accuracy (ppm)** | **Metabolite type** | **MS^2^ ions (ppm)** | **Lichen *S. glareosum*** |
| 105 | Loxodin | C_25_H_27_O_8_^-^ | 24.39 | 455.17114 | 455.17157 | 0.94 | D | 411.18173; 379.15619; 309.11307 | MeOH, LI-Cl, LI-MS, LI-TFB |
| 106 | Lobaric acid^a^ | C_25_H_27_O_8_^-^ | 24.63 | 455.17114 | 455.17194 | 1.76 | D | 411.17941; 367.19205; 352.16806; 296.10342 | MeOH, LI-Br, LI-Cl, LI-MS, LI-TFB |
| 107 | Unknown | C_28_H_25_O_8_^-^ | 25.31 | 489.15549 | 489.15439 | 2.25 | - | - | LI-TFB |
| 108 | Unknown | C_30_H_31_O_9_^-^ | 25.52 | 535.19736 | 535.19574 | 3.03 | - | - | LI-TFB |
| 109 | Unknown | C_19_H_23_O_2_^-^ | 25.87 | 283.17035 | 283.17004 | 1.09 | - | - | LI-Br, LI-Cl, LI-MS, LI-TFB |
| 110 | Usnic acid | C_18_H_15_O_7_^-^ | 26.15 | 343.08005 | 343.08255 | 7.29 | DBF | 328.05914; 259.06039; 231.06532 | MeOH, LI-Br, LI-Cl, LI-MS, LI-TFB |
| 111 | Haematommic acid lactone | C_9_H_5_O_4_^-^ | 26.27 | 177.01913 | 177.01900 | 0.73 | A | 133.02881; 119.04941; 105.03373; 163.03951 | MeOH, LI-Br, LI-Cl, LI-MS, LI-TFB |
| 112 | Atranorin^a^ | C_19_H_17_O_8_^-^ | 26.38 | 373.09289 | 373.09344 | 1.47 | d | 177.01880; 163.03951 | MeOH, LI-Br, LI-Cl, LI-MS, LI-TFB |
| 113 | Ethyl 4-O-methylolivetolcarboxylate | C_15_H_21_O_4_^-^ | 26.41 | 265.14453 | 265.14764 | 11.73 | A | 205.08659; 163.03935 | MeOH, LI-Br, LI-Cl, LI-TFB |
| 114 | Unknown | C_23_H_29_O_3_^-^ | 26.57 | 353.21222 | 353.21280 | 1.64 | - | - | LI-Br, LI-Cl, LI-MS, LI-TFB |
| 115 | Perlatolic acid | C_25_H_31_O_7_^-^ | 26.91 | 443.20753 | 443.20795 | 0.95 | d | 223.09680; 205.08653; 161.09654 | MeOH, LI-Cl, LI-MS, LI-TFB |
| 116 | Protoconstipatic acid derivative | C_21_H_29_O_5_^-^ | 27.05 | 361.20205 | 361.20273 | 1.88 | L | 317.21271; 299.2045 | MeOH |
| 117 | Norlobariol | C_23_H_27_O_7_^-^ | 27.48 | 415.17623 | 415.17554 | 1.66 | DE | 237.07614; 219.06625; 179.10722 | MeOH, LI-Br |
| 118 | Unknown | C_23_H_31_O_2_^-^ | 27.97 | 339.23295 | 339.23251 | 1.30 | - | - | LI-Cl, LI-MS, LI-TFB |
| 119 | 2,4-Dimethoxy-6-n-heptylbenzoic acid | C_16_H_23_O_4_^-^ | 29.29 | 279.16018 | 279.16342 | 11.61 | A | 235.1619; 180.03806 | LI-MS |
| ^a^ Identified by spiking experiments with an authentic compound. P = polyol, L = lipid, A = aromatic, D = depsidone, DE = diphenylether, d = depside, DBF = dibenzofuran. MeOH: Methanol extract; LI-Br: 1-butyl-3-methylimidazolium bromide extract; LI-Cl: 1-butyl-3-methylimidazolium chloride extract; LI-MS: 1-butyl-3-methylimidazolium methyl sulfate extract; LI-TFB: 1-butyl-3-methylimidazolium tetrafluoroborate extract. MS^2^: Daughter ions. | | | | | | | | | |

2. Figure 1. Chemical structures of the compounds identified by UHPLC-ESI-Q-Orbitrap-MS/MS.

Figure 1. *Cont.*

**Analysis of data**

Data adquisition was performed using MS-DIAL, ver. 4, and MATLAB (R2019b) software. The raw format data were first converted into “Analysis Base File” (ABF) format for treatment by MSDIAL. Then, peak spotting was performed by exploring retention time and accurate mass. MS-DIAL provides peak alignment of all samples and normalizes data based on TIC (total ion current).

Detection was based on calculated exact mass, and retention time of target compounds, as shown in Table 1. Then, it was compare with the daughter ion mass spectra (MS^2^) previously reported.

**3. Characterization data for lichen substances by using UHPLC/ESI/MS/MS**

All new compounds were tentatively detected in negative mode (Table 1).

*From [Bmim]MeSO_4_ extract (12 new compound tentatively identified)*

Peak 59 was identified as 2’,5-dimethoxyelatinic acid ([M – H]^−^ at m/z 445.1140). Its fragmentation produced a diagnostic MS ion at m/z 237.0404. This evidence allowed the methoxy group to be placed at C-5. To complete our structure, it was necessary to link position 2 to 5’, producing a depsidone that resembles elatinic acid.

Peak 62 was tentatively assigned as dehydroxyxy-2'-O-methylnorbarbatic acid. The main difference with 2'-O-methylnorbarbatic acid was the lack of OH group at C-2, which was indirectly evidenced by the fragments 179.07085; 163.03966.

Peak 73 was identified as 2’-methoxyglomellic acid. It was considered a derivative of glomellic acid (peak 68). The only difference was the presence of a methoxy group at C-2’ with the daughter fragment at m/z 251.0927 suggesting it.

Peak 74 was considered a derivative of peak 103. It was assigned as 4-demethyl-2’’-dehydrolobaric acid. The lack of methyl group at C-4 was indirectly evidenced by the fragment at m/z 223.0971 [C_12_H_15_O_4_]^−^.

Peak 81 resembles lobaric acid (peak 106) except for one methyl group at C-4. The fragment at m/z 193.0863 [C_11_H_13_O_3_]^−^ tentatively confirmed this proposal.

Peak 87 was identified as 5’-hydroxyglomellic acid (molecular anion at m/z 487.1620). The fragmentation of this peak produced ions at 253.0730 [C_12_H_13_O_6_]^−^, and 209.0814 [C_11_H_13_O_4_]^−^ confirming the presence of the OH group at C-5’ in this depside.

Peak 91 was tentatively identified as 2’’’-oxolividic acid, based on both its high resolution MS spectrum and a diagnostic daughter ion at m/z 237.0781. The presence of the carbonyl group at C-2’’’ was placed by biosynthetic considerations. The other daughter ions were not considered diagnostic ions since they only demonstrated the presence of carboxylic clusters.

*From [Bmim]BF_4_ extract (8 new compound tentatively identified)*

Peaks 33, 82, 103, 104, and 111 were previously identified in methanol extract, while peaks 62, 74, and 81 in [Bmim]MeSO4 extract were also detected.

*From [Bmim]Br extract (8 new compound tentatively identified)*

Peaks 33, 62, 74, 81, 82, 103, 104 and 111.

*From [Bmim]Cl extract (8 new compound tentatively identified)*

Peaks 33, 62, 74, 81, 82, 103, 104 and 111.

**4. UHPLC-ESI-Q-Orbitrap-MS/MS Chromatogram of the extracts of S. glareosum.**

**
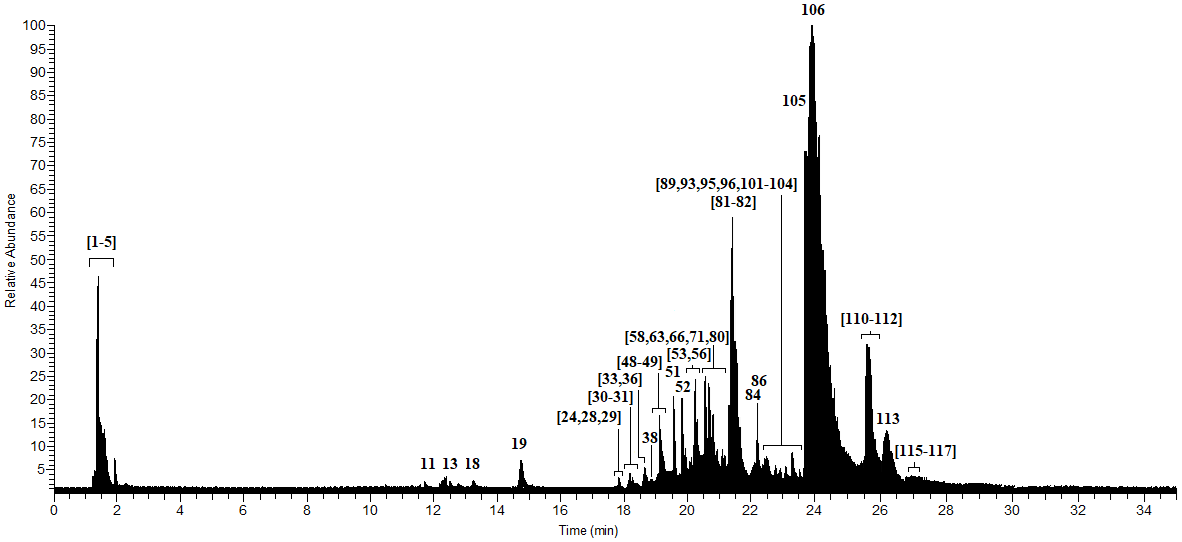
**

**Figure 1. UHPLC-ESI-MS-MS Chromatogram of methanolic extract of *S. glareosum*.**

**
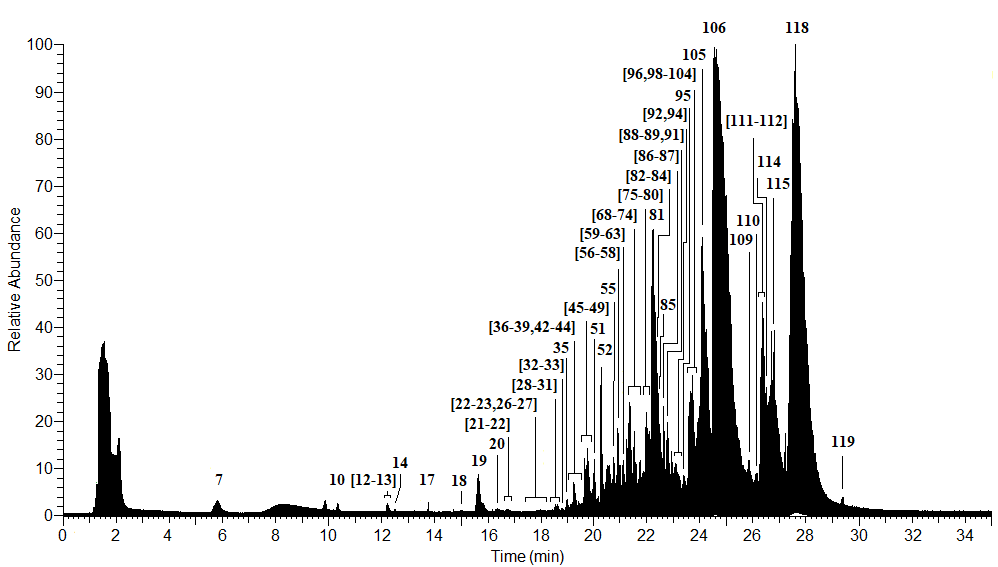
**

**Figure 2. UHPLC-ESI-MS-MS Chromatogram of 1-butyl-3-methylimidazolium methyl sulfate extract (LI-MS) of *S. glareosum*.**

**
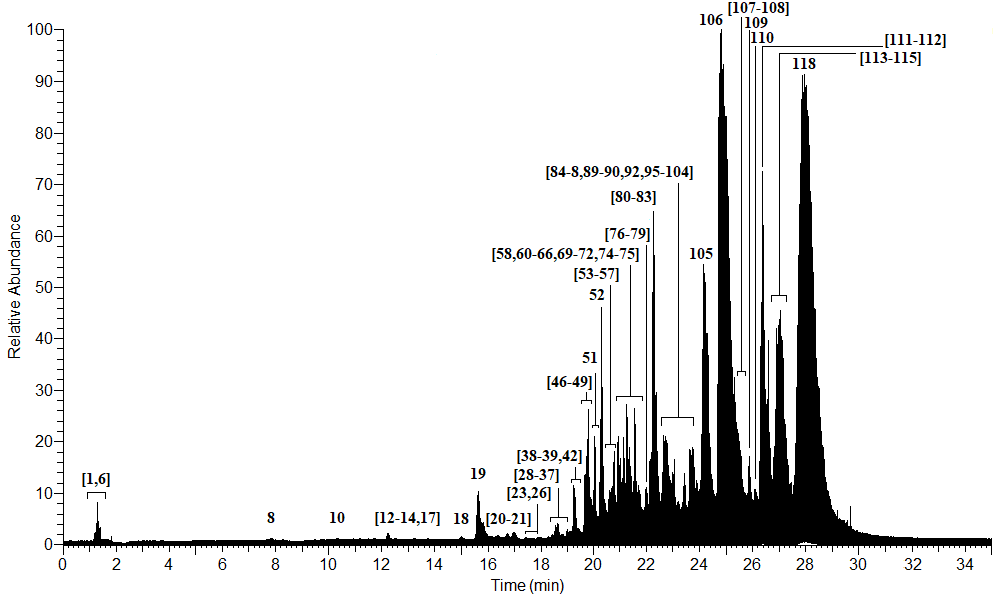
**

**Figure 3. UHPLC-ESI-MS-MS Chromatogram of 1-butyl-3-methylimidazolium tetrafluoroborate extract (LI-TFB) of S. glareosum.**

**
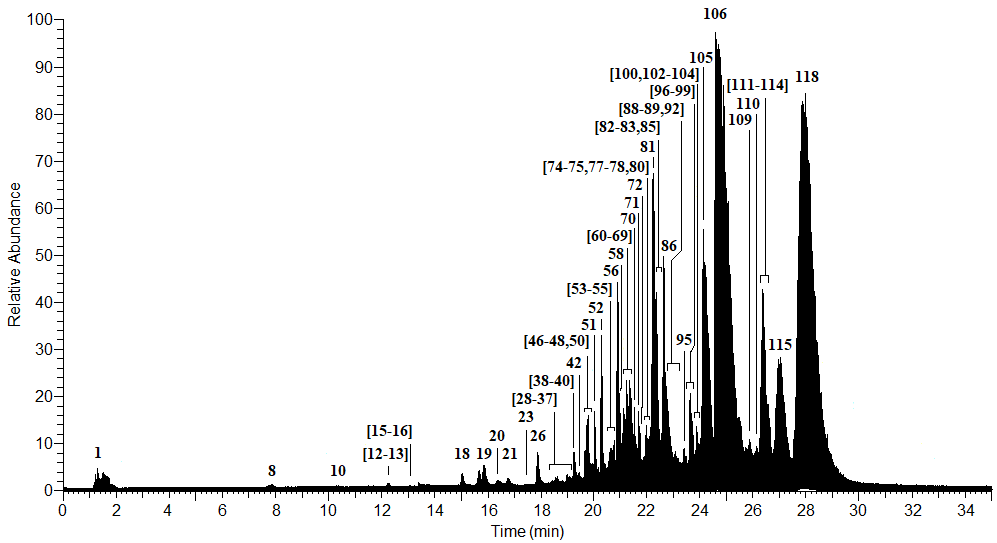
**

**Figure 4. UHPLC-ESI-MS-MS Chromatogram of 1-butyl-3-methylimidazolium chloride extract (LI-Cl) of S. glareosum.**

**
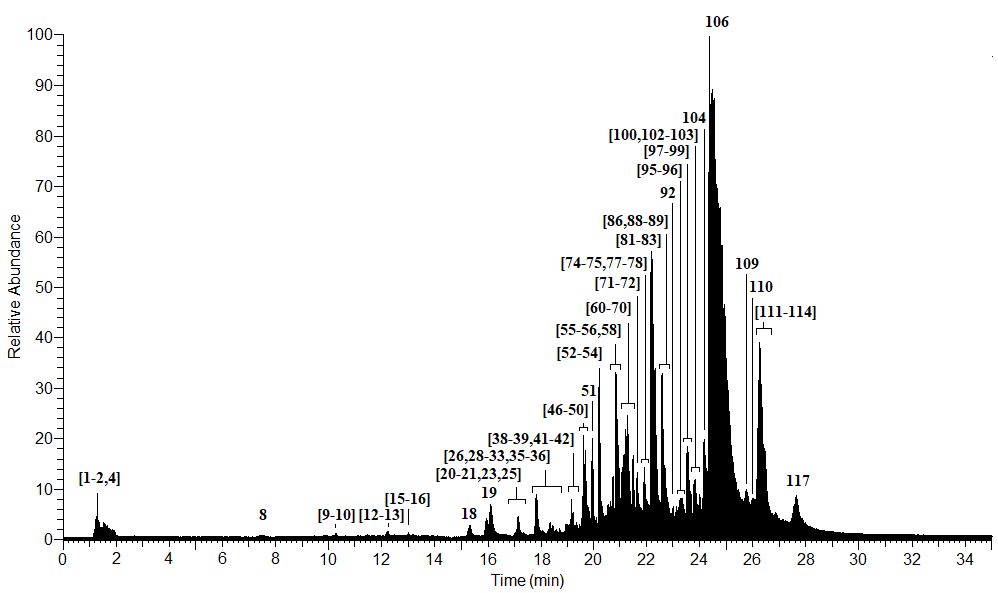
**

**Figure 5. UHPLC-ESI-MS-MS Chromatogram of 1-butyl-3-methylimidazolium bromide extract (LI-Br) of *S. glareosum*.**
